# Secondary bladder stone caused by delayed penetration of the bladder by a pubic fracture: A case report and literature review

**DOI:** 10.3892/etm.2024.12455

**Published:** 2024-02-27

**Authors:** Xinghao Wang, Xiao Guo, Zhiling Tang, Xiangjun Ying, Chenye Tang, Ruilin Shen

**Affiliations:** 1 Jiaxing University Master Degree Cultivation Base, Zhejiang Chinese Medical University, Hangzhou, Zhejiang 310000, P.R. China; 2 Department of Urology, The Second Hospital of Jiaxing, Jiaxing, Zhejiang 314000, P.R. China

**Keywords:** pubic fracture, ischemic necrosis, bladder injury, bladder stone, cystolithotomy

## Abstract

Pelvic fractures sometimes lead to injuries of the urinary bladder, which commonly present as gross hematuria, dysuria and lower abdominal pain. As a type of urinary stone, bladder stones are usually secondary to lower urinary tract obstruction, such as benign prostatic hyperplasia, urethral stricture, and neurogenic bladder. The present case report examines an unusual case of a delayed pubic fracture penetrating the bladder, which caused a secondary bladder stone. A 53-year-old man was first hospitalized at The Second Hospital of Jiaxing (Jiaxing, China) in January 2020 because of trauma-induced bleeding in the scalp and abdominal pain. The patient underwent abdominal exploration and partial bowel resection, and his condition stabilized after surgery. After discharge, the patient had regular outpatient check-ups every 2-3 weeks. However, after 3 months, in April 2020, the patient was readmitted to the hospital because of frequent urination, an urgent need for urination and dysuria. Abdominal computed tomography imaging and cystoscopy revealed a pubic fracture that had penetrated the bladder wall, accompanied by a bladder stone. Subsequently, cystolithotomy was performed, which provided significant relief of symptoms once the catheter was removed after 2 weeks. Since then, the patient has been followed up until January 2023 and had remained asymptomatic. Bladder stones caused by necrotic bone fragmentation are rare. Bladder injuries resulting from pelvic fractures can have delayed onset; therefore, clinicians should be aware of the possibility of urogenital injury in such patients. It is crucial for clinicians to comprehend the potential mechanisms involved, analyze the clinical data of patients, closely monitor their condition and implement appropriate treatment measures when necessary.

## Introduction

Urinary tract injury is a common complication associated with pelvic fractures. Previous studies have indicated that 7-25% of patients who sustain pelvic fractures, also suffer from concomitant damage to the urinary system, primarily affecting the bladder and urethra (1,2). However, the incidence of bladder injury (~6.8%) is notably lower compared with that of urethral injury (~9.9%) (3,4). Most bladder injuries occur at the time of the pelvic fracture or shortly after, when the urinary bladder ruptures due to laceration caused by a bone spur or compression stemming from the fracture itself. Pubic branch fractures are the predominant type of pelvic fractures that lead to bladder injury, accounting for ~89% of cases (5). Typical clinical manifestations of bladder injury include gross hematuria, dysuria and lower abdominal pain, accompanied by major urinary exosmosis (1). In cases of severe intraperitoneal bladder rupture, urine can permeate into the abdominal cavity, potentially causing life-threatening complications, such as peritonitis and sepsis (6). Consequently, prompt treatment is imperative upon the diagnosis of a ruptured bladder. In exceptional instances, however, bladder injuries may develop several days or weeks after the primary injury, rather than occurring at the time of injury. This is likely due to a secondary rupture at the site where a hematoma forms on the bladder wall (7). The current report presents another rare case in which a fragment originating from a delayed pubic fracture penetrated the bladder wall, thereby inducing bladder injury. Furthermore, the fragment dislodged into the bladder, resulting in a secondary bladder stone. The present case report serves as a reminder that delayed bladder injury, although extremely rare, can lead to certain complications and should not be disregarded in the management of patients with severe abdominal trauma.

## Case report

**Figure 1:**
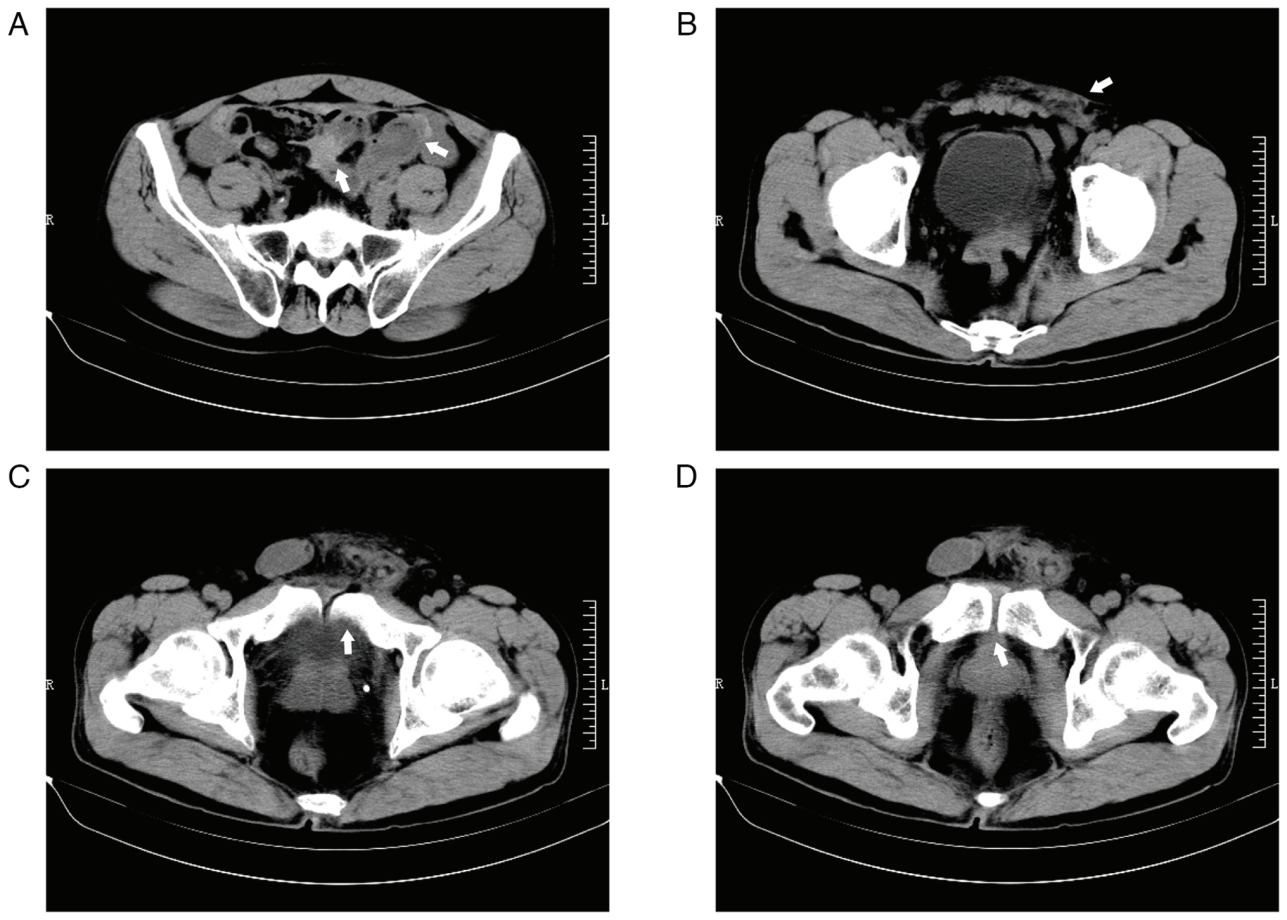
Abdominal injuries shown by axial computed tomography images (axial view; scale bar, 10 cm). (A) Injury to the small intestine and mesentery (arrows). (B) Injury to the left inguinal area (arrow). No apparent fracture was observed in (C) pubic rami (arrow) and (D) pubic symphysis (arrow). R, right; L, left.

A 53-year-old man was admitted to The Second Hospital of Jiaxing (Jiaxing, China) in January 2020, with scalp bleeding and abdominal pain caused by a road accident. The patient was knocked down by a car coming from the side while riding an electric bicycle, causing his left abdomen and groin area to be crushed by the falling bike. Physical examination at that time revealed multiple abrasions on the body and bruising and swelling in the left groin area. Emergency plain abdominal computed tomography (CT) imaging showed injuries to the small intestine, mesentery and the left inguinal area without any apparent signs of pelvic injury (Fig. 1). Subsequently, the patient underwent exploratory laparotomy, which revealed a severe rupture of a portion of the small intestinal lumen and laceration of the mesentery of both the small intestine and sigmoid colon, accompanied by active bleeding. As a result, a section of the small intestine was removed through open surgery, and the lacerated mesentery was repaired. A total of 2 weeks later, the patient had recovered well and was discharged from the hospital. By the time of discharge, the patient's abdominal pain was notably relieved, and there were no urinary symptoms, such as hematuria and painful, frequent or urgent urination. The surgical incision healed properly, and the swelling in the left inguinal area subsided.

**Figure 2:**
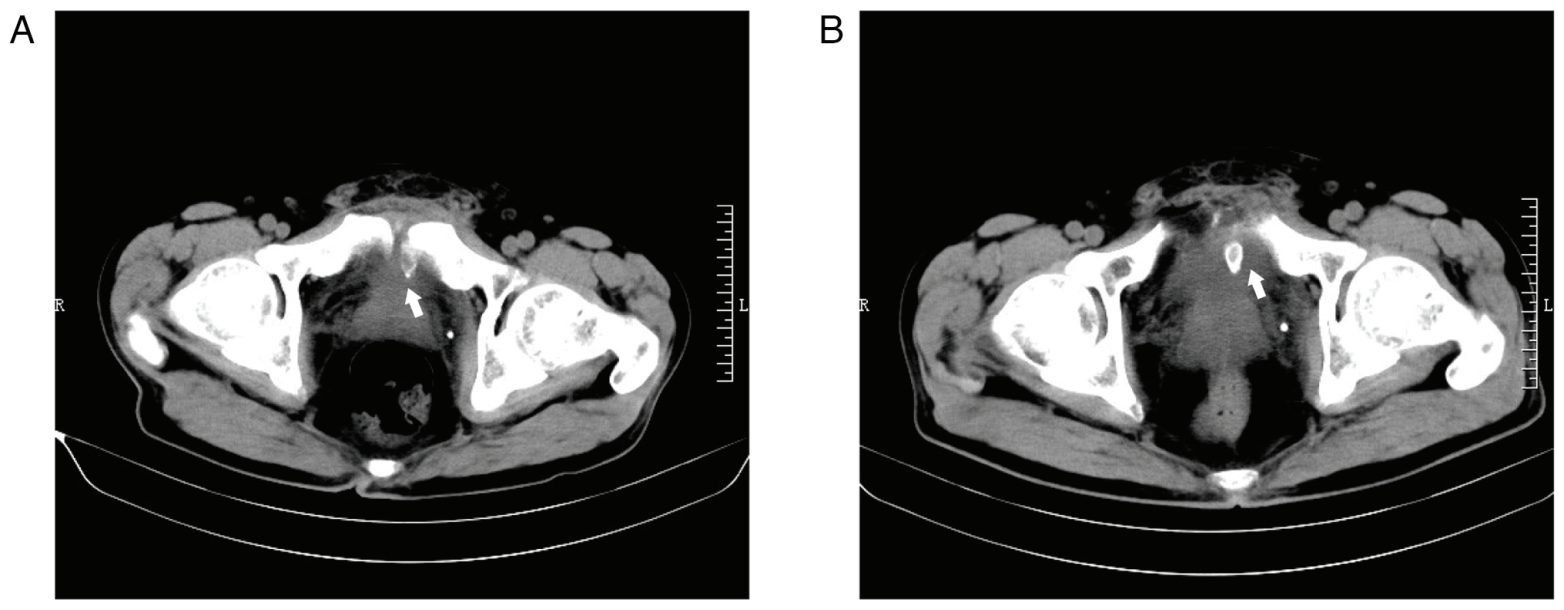
Delayed left pubic fracture was visible in the patient's computed tomography images during follow-up after discharge (axial view; scale bar, 10 cm). (A) A fracture of the left pubic bone was found 5 weeks after the injury (arrow). (B) The fragmented bone migrated closer to the bladder area at 7 weeks after the injury (arrow). R, right; L, left.

To assess the recovery of the patient's intestinal injury, an abdominal CT scan was performed at the outpatient clinic of The Second Hospital of Jiaxing 5 weeks after the injury (February 2020), which incidentally revealed a fracture of the left pubic bone and a fragmented bone appearance in the posterior region of the pubic symphysis (Fig. 2A). A total of 7 weeks after the injury (March 2020), another CT scan showed a more pronounced fracture, and that the dissociative bone had shifted closer to the bladder area (Fig. 2B). As there was no evident local pain or discomfort, close monitoring was maintained at that time.

**Figure 3:**
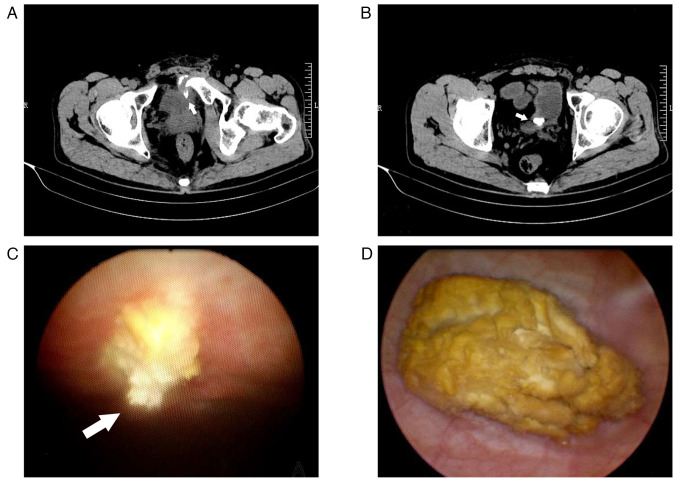
Left pubic fracture and secondary bladder stone observed 12 weeks after the injury. Axial computed tomography scan revealed that (A) the fragmented bone had penetrated the left anterior wall of the bladder (arrow) and (B) a stone measuring ~15x10 mm was present within the bladder (arrow) (axial view; scale bar, 10 cm). Optical images captured during the flexible cystoscopy revealed that (C) the bone fragment from the left pubis had penetrated the left anterior wall of the bladder (arrow) and (D) an osseous stone with a dark yellow color and loose consistency was discovered inside the bladder. R, right; L, left.

**Figure 4:**
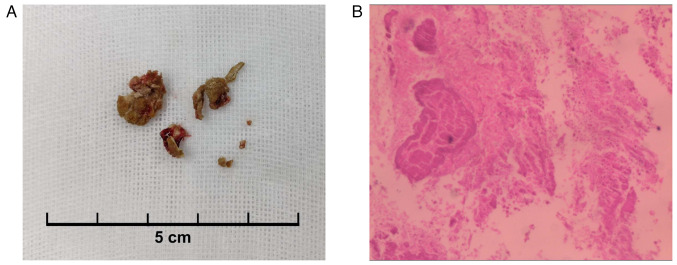
Specimen and pathological staining of the bladder stone. (A) Bladder stone specimen removed during the operation (scale bar, 5 cm). (B) Hematoxylin and eosin staining confirmed the presence of abundant eosinophilic amorphous substance, consistent with necrotic bone (magnification, x200).

A total of 12 weeks after the injury (April 2020), the patient began experiencing symptoms of frequent, urgent and painful urination, but there was still no macroscopic hematuria. Abdominal CT revealed that the previously fragmented bone in the posterior area of the pubic symphysis had penetrated the left anterior wall of the bladder, although it appeared smaller compared to its size in the CT scan performed at 5 or 7 weeks after the injury (Fig. 3A). In addition, a 15x10 mm stone was found in the bladder (Fig. 3B). Therefore, the patient was readmitted to the hospital and underwent a flexible cystoscopy, which revealed that the bone fragment from the left pubic bone had penetrated the left anterior wall of the bladder (Fig. 3C). In addition, a dark yellow, loose and osseous stone was found inside the bladder (Fig. 3D), consistent with the CT imaging findings. Due to the attachment of the pubic bone fragment to the anterior wall of the bladder, it was not possible to separate it from the bladder wall or repair the damaged area by endoscopic treatment. Therefore, an open surgery was performed in April 2020. During the operation, the bladder stone was successfully removed (Fig. 4A). The bladder wall at the site of the injury was adequately separated from the fractured end of the pubic bone and repaired. Furthermore, the fractured end of the pubic bone was smoothed to prevent its re-penetration into the bladder. The removed bladder stone was sent for pathological examination. It was fixed in 10% neutral formalin at room temperature for 24 h and subsequently embedded in paraffin. A 4-µm thick section was prepared and deparaffinized using xylene, rehydrated with gradient of alcohol solutions, and then subjected to hematoxylin staining for 10 min followed by eosin staining for 10 sec at room temperature. The section was reviewed and photographed under an optical microscope, confirming the presence of abundant eosinophilic amorphous substance, consistent with necrotic bone (Fig. 4B). The patient had a smooth recovery after the operation. When the catheter was removed, after 2 weeks, the symptoms of frequent, urgent and painful urination were alleviated.

**Figure 5:**
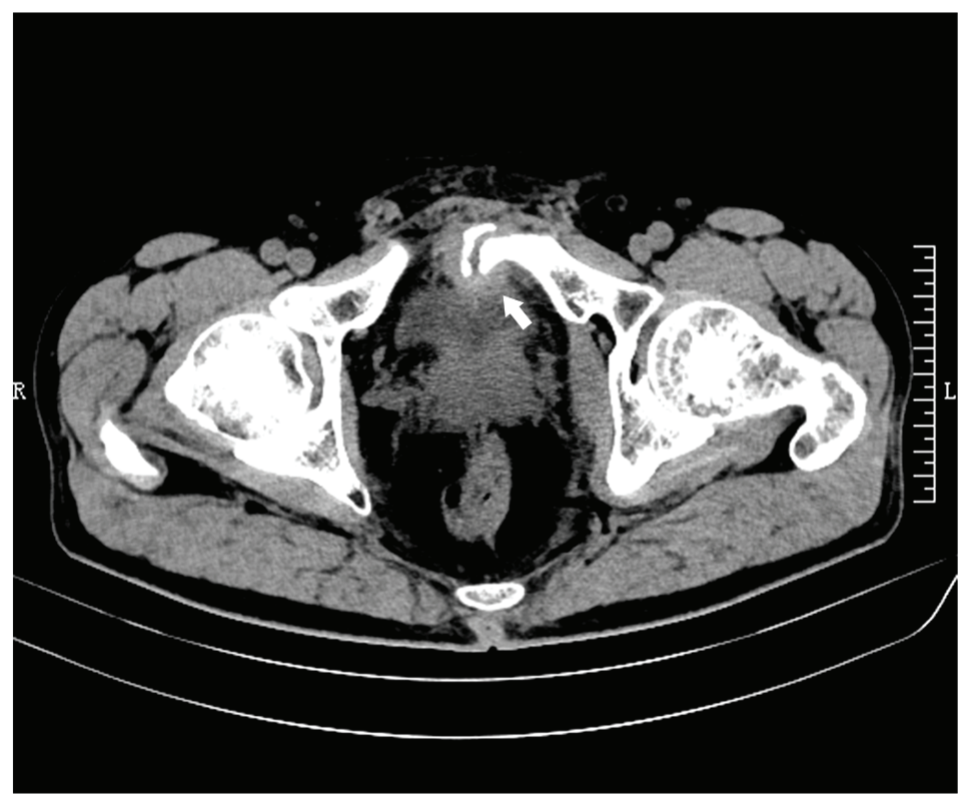
Recovery of the bladder 7 months after the operation. Computed tomography scan showed that the left pubic bone was closely situated near the anterior wall of the bladder, without any evidence of bone fragments penetrating the bladder wall or the formation of bladder stones (arrow) (axial view; scale bar, 10 cm). R, right; L, left.

A total of ~7 months after the surgery (November 2020), the patient's abdominal CT scan showed a satisfactory recovery of the bladder. The left pubic bone was closely situated near the anterior wall of the bladder, without any evidence of bone fragments penetrating the bladder wall or the formation of bladder stones (Fig. 5). The patient no longer experienced symptoms involving frequent and urgent urination or pain during urination. The patient exhibited a smooth recovery with no signs of recurrence during the follow-up period until January 2023.

## Discussion

Blunt trauma accounts for 60-85% of bladder injuries, and traffic accidents are one of the most common causes, contributing to ~41% of cases (8-10). In the present case, the bladder injury was also related to a traffic accident; however, it did not occur immediately, at the time of the accident, but rather developed with a delay. In summary, the traffic accident the patient suffered led to injuries in the left abdomen and inguinal region, with minor damage to the left pubic bone. Over the 3 months following the accident, a trauma-induced fracture occurred in the left pubic bone, causing a fragment to gradually puncture the bladder wall and eventually leading to the formation of a stone inside the bladder. To the best of our knowledge, no similar case has been previously reported.

Bladder stones constitute ~5% of all urinary stones (11) and occur with a relatively higher prevalence in men (0.016-0.022%), although the incidence has decreased over time (12). Clinically, the majority of bladder stones are secondary in nature and result from upper urinary tract stones, lower urinary tract obstruction, recurrent infections or the presence of foreign bodies in the bladder (11). Various types and sources of foreign bodies can be found in the bladder, including exogenous and endogenous ones. A number of foreign bodies are associated with sexual stimulation, psychological disorders and other mental disorders. Examples include glass rods, thermometers, pencils, cotton swabs and other items used by patients who are seeking stimulation. These objects are inserted into the bladder through the urethra, leading to inflammatory reactions, gradual calcification and the subsequent formation of bladder stones (13,14). Schmitt *et al* (15) reported a case where artificial fishing worms were discovered in the urinary bladder 3 years after their initial insertion, which had transformed into bladder stones. In addition to the aforementioned exogenous foreign bodies, bladder stones may also develop when surgical dressings are inadvertently left in the bladder (16). Another possible cause of bladder stones is the migration of foreign bodies from adjacent organs into the bladder. There have been cases reported where intrauterine devices had caused perforation of the uterus, followed by penetration of the bladder, which resulted in the presence of a foreign body within the bladder and the formation of bladder stones at varying levels (17-19). In the current patient, the bladder stone formed when a piece of the pubic fracture penetrated the bladder and gradually detached due to ischemia and necrosis. Bladder stones of this nature and origin are rare. In the present case, the pelvic fracture was not dealt with initially or soon after the injury because the imaging and clinical findings did not suggest a rupture of the bladder. However, the follow-up CT scan at 3 months after the injury revealed the signs of bladder injury and a bladder stone, which required surgical treatment. Therefore, in cases of rare, delayed bladder injuries caused by pelvic fractures, only close follow-up can ensure timely diagnosis and appropriate treatment.

The mechanism of the secondary bladder injury following the pelvic fracture was also investigated in the current patient. Bladder injuries can occur not only through direct penetration or compression by fracture pieces, but also via indirect force transmitted through ligaments (20). In rare instances, bladder entrapment can also cause such injuries (20). Tolkach *et al* (21) reported an unusual case in which bladder entrapment occurred at the fracture site several months after an acetabular fracture, resulting in formation of a fistula and urinary leakage, as well as a stone in the hip joint cavity. The present case is similar to that of Tolkach *et al* (21), as the patient also presented with noticeable urinary symptoms ~3 months after the pelvic fracture. However, the distinctive feature of the current case is that the bladder was compressed by the fractured tip of the pubic bone, which eventually necrotized and detached after piercing the bladder, thereby leading to formation of a rare bladder stone composed of necrotic bone material. The mechanism of the delayed penetration of the pubic fragment into the bladder may be as follows: On the one hand, the pelvic fracture remained unstable during movements, continuously exerting pressure on the bladder and causing local ischemia and mechanical injury; on the other hand, as urine accumulated, the pressure within the bladder increased, pushing the bladder wall against the sharp tip of the pubic fracture, causing bladder injury.

The rate of missed diagnosis of associated bladder and urethral injuries in the initial evaluation of pelvic fractures at the time of occurrence is as high as 23% (1,22). Cystography, including plain film and CT scan, is an important diagnostic method for bladder injury. CT is particularly useful in detecting kidney and other abdominal organ injuries. Pelvic fracture accompanied by gross hematuria is an absolute indication for immediate cystography if the patient is hemodynamically stable; while an isolated pelvic fracture or hematuria (microscopic or macroscopic) should only be considered a relative indication for cystography and must be associated with the patient's clinical signs, including suprapubic tenderness, abdominal hematoma, perineal and upper thigh edema, pelvic effusion, inability to urinate, and increased blood urea and creatinine levels (23-26). The risk of bladder injury in patients with pelvic fractures exhibiting only microscopic hematuria is <1% according to the American Urological Association (AUA) Core Trauma Guidelines (8). Hypotension is an important indicator of severe urinary tract injury (5). However, there has been a case in which bladder injury was confirmed during surgery despite negative cystography results (27). The causes of false negative cystography in such cases may include failing to obtain a lateral-view or excretory X-ray urography and the inadequate distention of the bladder (<400 ml) (22). In case of insufficient bladder distention, small bladder ruptures may be blocked by the omentum or blood clots, leading to false negative cystography results (25). Furthermore, the AUA points out that certain types of fractures, such as pubic symphysis separation and obturator ring fracture displacement >1 cm, indicate potential bladder injury (26). However, it is important to note that bladder rupture, especially intraperitoneal rupture, cannot be excluded if the patient does not have a pelvic fracture.

The British Association of Urological Surgeons recommends that bladder injury should be further classified as intraperitoneal, extraperitoneal or a combination of the two. Additionally, injuries to the ureter and urethra should be considered (28). The AUA recommends using Foley catheter drainage in patients with uncomplicated extraperitoneal bladder injury. However, surgical exploration and repair should be performed in patients with intraperitoneal bladder rupture or complicated extraperitoneal bladder injury. It is important to conduct postoperative follow-up to confirm healing of the bladder injury through cystography (26).

In conclusion, delayed bladder injury caused by pelvic fractures is uncommon; however, it should not be overlooked and warrants careful consideration. Dealing with such cases necessitates close collaboration among orthopedic surgeons, urologists and trauma surgeons. Clinicians must be trained to consider genitourinary system injuries when diagnosing and treating patients with pelvic fractures. This involves understanding the potential mechanisms involved, thoroughly analyzing the patients' symptoms and imaging data, and closely monitoring their progress. Upon diagnosis, appropriate treatment measures should be promptly taken.

## Data Availability

The data generated in the present study may be requested from the corresponding author.
